# Correspondence

**Published:** 1902-03

**Authors:** 


### CORRESPONDENCE.

OFFICE OF GOVERNMENT INSPECTOR OF STOCK,
ANDREW SMITH, F.R.C.V.S., Inspector.
TORONTO, 1902.

EDITOR OF THE JOURNAL OF COMPARATIVE MEDICINE, ETC.

DEAR SIR: In the January number of your excellent JOURNAL, which has just come to hand, I notice an article by Mr. V. P. Smith, V.S., who graduated at the Chicago Veterinary College in 1895, and who attended the Ontario Veterinary College a few years previously. He describes a case of "Dropsy of the Amnion" that he has met with in his practice, and says that during his attendance at these two colleges he never heard this condition mentioned, and that he has no literature on the subject.

Lectures on veterinary obstetrics, in which "dropsy of the amnion" is fully explained, and of which I have seen several cases in both the mare and in the cow, were given by myself in the Ontario Veterinary College at the time Mr. V. P. Smith attended the college, and are still continued, and I must express my regret that he did not hear them.

I have also always been particular in drawing the attention of the students to the distinction that must be drawn between "dropsy of the amnion" and "dropsy of the uterus," which is not uncommon in the cow and heifer.

Both these conditions are well described in Fleming's *Veterinary Obstetrics*, which was published about the year 1877. It is one of the text-books of the Ontario Veterinary College, and much useful information may be obtained from it. There was a new edition of this valuable work published in 1895. I would strongly advise Mr. Smith to add it to his library, as he says that he has no literature on the subject.

C. HEATH SWEETAPPLE,
Professor of Cattle Pathology and Veterinary Obstetrics,
Ontario Veterinary College, Toronto, Canada.

CHICAGO, March 6, 1902.

EDITOR OF THE JOURNAL OF COMPARATIVE MEDICINE, ETC.

DEAR DOCTOR: In the January issue of the JOURNAL, page 49, we note a letter from Dr. J. P. Foster, State Veterinarian of South Dakota, criticising our literature in regard to mallein, or, at all events, so far as the claims made for mallein as a curative agent for glanders.

Dr. Foster states that he has used about 400 doses of our mallein as a diagnostic agent, and concedes that it is of value in that respect; but he condemns us for claiming any curative properties for mallein, this condemnation being based not upon his own experience, but upon the experience of a single layman who obtained some mallein (make not stated) and did not find that it effected a cure. Perhaps it is unnecessary to comment upon Dr. Foster's conclusion except to quote Dr. Law's words, published on page 247 of your April, 1901, issue, namely: "Your correspondent, J. P. Foster, makes a statement which is by no means justified by the facts, and which should be corrected in the interest alike of truth and of veterinary education."

Dr. Foster asks if you or any of your readers can inform him if any of the best authorities on veterinary science concede that mallein is a cure for glanders. As I have been a diligent reader of your JOURNAL and other veterinary literature for a number of years, perhaps I may be permitted to reply to this question. Perhaps it will be conceded that Dr. Nocard, the well-known French veterinarian, is an authority upon veterinary science; and perhaps it will also be conceded that Dr. Roux, of the Paris Pasteur Institute, and who has been connected with the Pasteur laboratories for the last thirty years, is an authority upon comparative medicine.

These two authorities have experimented with mallein as a curative agent for glanders, and have publicly stated that mallein is a cure for glanders. The result of their experiments was set forth in the March, 1896, issue of the *Recueil de Medecine Veterinaire*, published in Paris, France, by the Faculty of the celebrated Alfort Veterinary College.

About the same time I received from the Pasteur Institute a communication on the subject, which communication I transmitted to the American veterinary journals, and it was published—at all events in the *American Veterinary Review*, in June, 1896; but I am unable to lay my hands upon its publication in your own JOURNAL.

Although I have seen several reports from veterinarians who claim to have cured several cases of glanders by the employment of mallein, yet I would refer to the report of Dr. W. L. Langdon, of Omaha, Neb., published on page 246 of your issue of April, 1901. It seems to me that if Dr. Foster had read Dr. Law's remarks above quoted he could not have failed to have read Dr. Langdon's report, as it stares a reader in the face.

We are not engaged in the "sure cure" or even in the "sure preventive" business, and our connection with the Pasteur Institute and our own reputation as a commercial concern would not permit of our making claims that were not well-founded. We plead not guilty to Dr. Foster's charge of being "almost criminal." Perhaps it is unnecessary for us to comment upon Dr. Foster's suggestion that the "Pasteur people," like Roux and Nocard, are "on a par with the horse book," or, in other words, with the so-called quack medicine people. The extenuating feature in Dr. Foster's communication is that he admits that he may be mistaken, and we would therefore respectfully submit that instead of jumping to a conclusion from the experience of a layman he conduct his tests himself in accordance with the experience of Drs. Roux and Nocard, as already published. It would be a joke on Dr. Foster if it were found that the horses treated by Dr. Foster's layman friend swallowed the thermometer, or perhaps the injection of mallein was *per rectum*. Pardon this levity, but I have before me page 52 of your January issue.

I trust that you will give this letter the same prominence which you have given to that of Dr. Foster.

Yours truly,

HAROLD SORBY,

General Manager Pasteur Vaccine Company.

